# The Pandemic of Coronary Heart Disease in the Middle East and North Africa: What Clinicians Need to Know

**DOI:** 10.1007/s11883-023-01126-x

**Published:** 2023-08-24

**Authors:** Yosef Manla, Wael Almahmeed

**Affiliations:** Department of Cardiology, Heart, Vascular and Thoracic Institute, Cleveland Clinic Abu Dhabi, Abu Dhabi, United Arab Emirates

**Keywords:** Coronary heart disease, Middle East, North Africa, Hypertension, Diabetes mellitus, Hyperlipidemia

## Abstract

**Purpose of Review:**

Coronary heart disease (CHD) is the leading cause of morbidity, mortality, and disability in the Middle East and North Africa (MENA). While the prevention, diagnosis, and management of CHD have been detailed in international guidelines, we aimed in this review to quantify the pandemic of CHD in the MENA region and highlight regional patient characteristics, clinical challenges, and future directions to optimize CHD care in the region.

**Recent Findings:**

Patients with CHD in the MENA feature younger age at presentation and worse prognosis in women. Despite the high burden of CHD risk factors, many of these factors remain underrecognized, undertreated, and uncontrolled. Additionally, CHD care is hampered by poor patient awareness, inefficient preventive strategies, and limited access to guideline-recommended therapeutics.

**Summary:**

All stakeholders involved in healthcare should work together to develop and execute strategies aimed at tackling the burden of CHD in the MENA.

## Introduction

The Middle East and North Africa region (MENA, referred to as North Africa and Middle East in the Global Burden of Disease (GBD) study) is one of the seven super regions of the GBD study. It encompasses 21 countries, and it had an estimated population of 608.7 million in 2019 (Table 1) [1, 2]. The region features cultural, religious, political, and socio-economic variations among its countries [3]. In 2019, the socio-demographic index (SDI), a composite indicator of development status that strongly correlates with health outcomes (ranges from 0 to 1), measured 0.66 for the MENA region and ranged between 0.34 (Afghanistan) and 0.88 (United Arab Emirates) (Table 1) [4]. In addition, several MENA countries are war-affected, significantly impacting healthcare delivery and outcomes [5]. Among the GBD seven super regions, the MENA region has the highest age-standardized rates of coronary heart disease (CHD, referred to as ischemic heart disease in the GBD study) incidence and prevalence, and the second highest age-standardized rate of mortality and disability-adjusted life years (DALY, defined as the sum of the years of life lost due to premature mortality and the years lived with a disability due to prevalent cases of the disease) [6]. While the prevention, diagnosis, and management of CHD have been detailed in international guidelines, we aimed in this review to quantify the pandemic of CHD in the MENA and highlight regional patient characteristics, clinical challenges, and future directions to optimize CHD care in the region (Fig. 1).

**Table 1:** Estimates of the Global Burden of Disease study on the incidence, prevalence, deaths, and disability-adjusted life years due to coronary heart disease alongside socio-demographic and health coverage indices for the MENA countries in 2019

Country	Socio-demographic index (0–1)*	Health coverage index (0–100)**	CHD incidence***	CHD prevalence***	CHD deaths***	DALY due to CHD^#,^***
North Africa and Middle East	0.66	–	2,550,432 (2,287,730–2,826,390)	19,979,927 (18,501,725–21,563,635)	799,484 (706,349–909,787)	17,994,822 (15,580,582–20,811,862)
Afghanistan	0.34	37	79,901 (71,092–89,426)	580,638 (535,688–628,880)	34,628 (26,989–42,665)	984,230 (750,693–1,275,467)
Algeria	0.65	75	179,174 (159,671–199,669)	1,447,170 (1,337,136–1,562,325)	58,692 (47,673–71,563)	1,167,612 (927,774–1,464,819)
Bahrain	0.75	71	6,461 (5,475–7,566)	49,534 (45,552–54,048)	863 (698–1,060)	23,147 (18,662–28,406)
Egypt	0.66	70	450,548 (411,983–491,750)	3,263,746 (3,033,581–3,513,428)	181,885 (138,959–233,632)	4,419,938 (3,313,613–5,806,684)
Iran	0.67	77	593,001 (515,830–676,050)	4,335,510 (3,950,113–4,761,788)	102,799 (94,455–111,215)	2,025,424 (1,898,318–2,228,261)
Iraq	0.67	55	140,746 (125,823–156,094)	1,141,916 (1,054,979–1,233,916)	46,848 (38,263–55,511)	1,070,932 (845,995–1,307,890)
Jordan	0.73	60	37,937 (32,682–43,525)	310,388 (286,477–336,917)	6,111 (5,196–7,286)	147,481 (124,436–177,498)
Kuwait	0.85	70	16,837 (14,644–19,359)	136,260 (125,999–147,518)	2,599 (2,165–3,105)	71,677 (59,489–86,115)
Lebanon	0.71	72	32,025 (28,355–35,958)	257,386 (238,945–276,978)	12,251 (8,866–14,092)	233,372 (171,955–270,077)
Libya	0.71	60	29,205 (25,487–33,143)	234,280 (217,472–253,277)	7,827 (6,177–10,363)	183,159 (144,662–247,389)
Morocco	0.55	73	190,736 (173,097–211,943)	1,530,983 (1,412,023–1,666,262)	72,012 (56,906–84,501)	1,551,023 (1,191,649–1,885,658)
Oman	0.78	69	12,346 (10,824–14,055)	92,282 (85,361–100,197)	3,412 (3,046–3,828)	83,213 (72,529–95,690)
Palestine	0.59		13,978 (12,080–16,022)	108,744 (100,339–117,400)	3,810 (3,326–4,366)	86,705 (75,038–99,657)
Qatar	0.83	74	6,440 (5,456–7,538)	46,196 (42,121–50,305)	830 (631–1,068)	24,220 (18,355–31,467)
Saudi Arabia	0.81	73	108,673 (96,041–122,753)	835,249 (771,862–903,746)	29,689 (24,089–36,176)	883,559 (702,673–1,099,540)
Sudan	0.52	44	113,150 (101,102–126,115)	880,414 (812,354–950,643)	43,187 (33,613–54,936)	1,019,494 (771,849–1,347,945)
Syrian Arab Republic	0.62	56	80,332 (71,888–89,818)	586,347 (544,902–633,879)	33,542 (26,239–43,170)	765,019 (586,984–1,001,672)
Tunisia	0.67	70	68,220 (60,187–77,244)	546,939 (506,332–589,711)	21,457 (16,189–27,281)	408,688 (304,296–531,700)
Turkey	0.75	79	281,417 (252,886–312,699)	2,785,049 (2,532,630–3,082,413)	99,046 (80,454–120,867)	1,847,044 (1,491,707–2,245,824)
United Arab Emirates	0.88	78	27,614 (23,612–32,368)	197,600 (180,763–216,933)	4,880 (3,504–6,755)	174,392 (124,284–243,500)
Yemen	0.41	44	79,100 (70,449–88,561)	592,996 (547,709–641,372)	32,305 (26,020–42,118)	806,211 (620,074–1,081,163)

*CHD* coronary heart disease, *DALY* disability-adjusted life years

*Socio-demographic index is the geometric mean of 0 to 1 indices of total fertility rate under the age of 25, mean education for those ages 15 and older, and lag distributed income per capita. As a composite, a location with an SDI of 0 would have a theoretical minimum level of development relevant to health, while a location with an SDI of 1 would have a theoretical maximum level (4). **Universal Health Coverage index is defined as the average coverage of essential services based on tracer interventions that include reproductive, maternal, newborn, and child health, infectious diseases, non-communicable diseases, and service capacity and access among the general and the most disadvantaged population. Reported on a unitless scale of 0 to 100, which is computed as the geometric mean of 14 tracer indicators of health service coverage (28). ***Data source: the Global Burden of Disease Study 2019 (6). Data were represented as estimates (95% uncertainty interval)

#The sum of the years of life lost due to premature mortality and the years lived with a disability due to prevalent cases of the disease

**Fig. 1:**
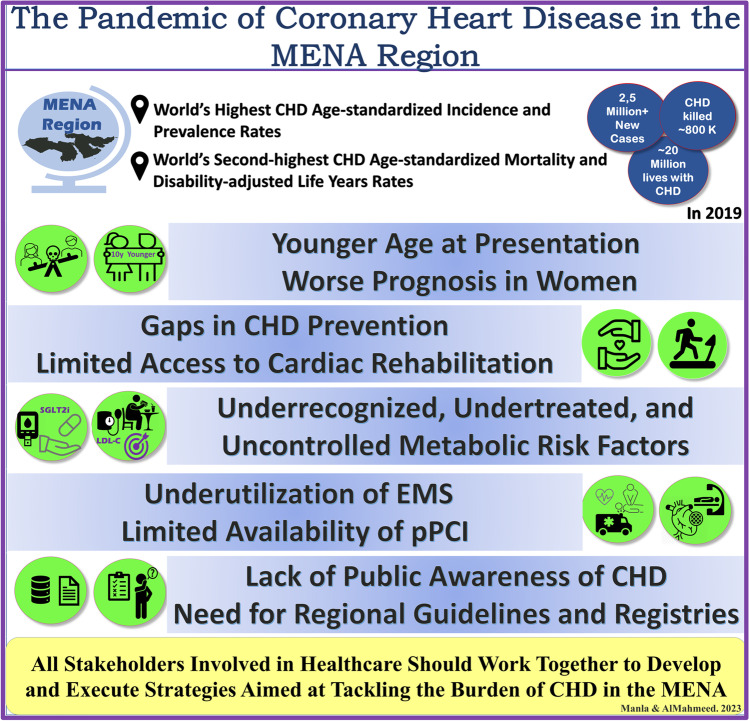
The Pandemic of CHD in the MENA: epidemiology and challenges. Abbreviations: CHD coronary heart disease, EMS emergency medical services, MENA Middle East and North Africa, pPCI primary percutaneous coronary intervention, SGLT2i sodium-glucose co-transporter-2 inhibitors

## The Pandemic of Coronary Heart Disease in the MENA

### The Incidence of CHD in the MENA

**Fig. 2:**
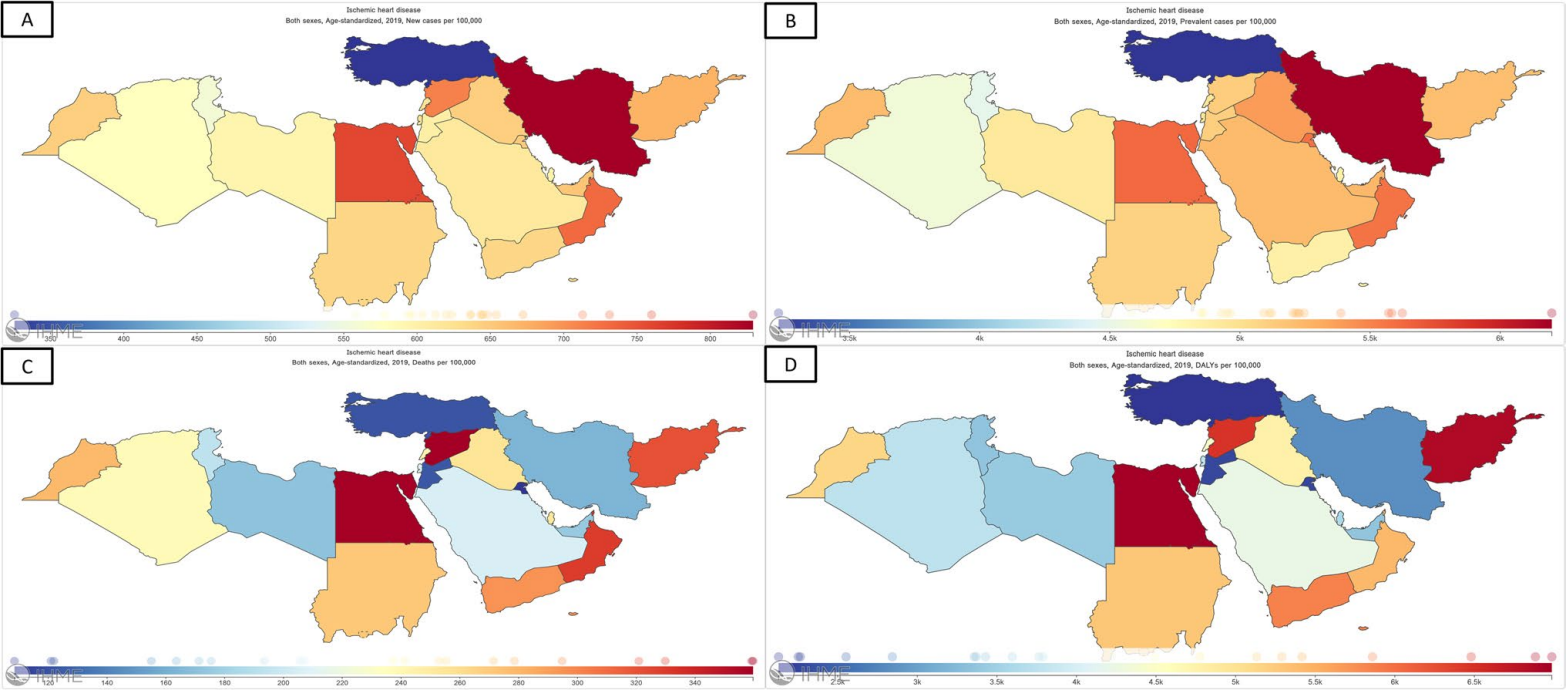
Estimates on the age-standardized rates of coronary heart disease incidence (**A**), prevalence (**B**), deaths (**C**), and disability-adjusted life years (**D**) in the MENA region in 2019. (Source: The Global Burden of Disease Compare Tool, with permission from: Institute for Health Metrics and Evaluation [7])

Within the seven GBD super regions, the highest age-standardized rate of CHD incidence was recorded in the MENA [6]. CHD contributed the most to the number of incident cases of cardiovascular disease (CVD) in the region (58.5%), with 2,550,431.6 uncertainty interval (UI) (2,287,729.8–2,826,389.56) cases (Table 1), corresponding to an increase of 33% in the rate of incidence (314.4 to 419 cases per 100,000) and a decrease of 9% (674.5 to 613.9 cases per 100,000) in the age-standardized rate of incidence between 1990 and 2019. At a national level, in 2019, the three countries with the highest age-standardized incidence rates were Iran (829.1 [UI 719.9–945.2] per 100,000), Egypt (759.9 [UI 705.9–819.4] per 100,000), and Oman (731.2 [UI 639.4–831.3] per 100,000), while the three countries with the lowest age-standardized incidence rates were Turkey (325.5 [UI 293.7–359.9] per 100,000), Tunisia (558.1 [UI 496.6–627.1] per 100,000), and Algeria (577.8 [UI 525.2–637.5] per 100,000). Figure 2A shows the variation in CHD age-standardized incidence across the MENA in 2019 [7].

### The Prevalence of CHD in the MENA

Within the seven GBD super regions, the highest age-standardized rate of CHD prevalence was recorded in the MENA [6]. CHD contributed the most to the number of prevalent cases of CVD in the region (54.7%), with 19,979,927.2 (UI 18,501,725.4–21,563,634.7) cases (Table 1), corresponding to an increase of 41% in the rate of prevalence (2,333 to 3,282.3 cases per 100,000) and a decrease of 3% (5,087.4 to 4,911.1 cases per 100,00) in the age-standardized rate of prevalence between 1990 and 2019 [6]. At a national level, in 2019, the three countries with the highest age-standardized prevalence rates were Iran (6,198.5 [UI 5,644.4–6,814.6] per 100,000), Egypt (5,623.95 [UI 5,255.3–6,014.9] per 100,000), and Kuwait (5,583.1 [UI 5,166.6–6,015.9] per 100,000), while the three countries with the lowest age-standardized prevalence rates were Turkey (3,227 [UI 2,942.1–3,563.6] per 100,000), Tunisia (4,480.2 [UI 4,161–4,823.8] per 100,000), and Algeria (4,581.4 [UI 4,248.9–4,938.7] per 100,000) [6]. Figure 2B shows the variation in CHD age-standardized prevalence across the MENA [7].

### CHD Mortality and its Attributable Risk Factors in the MENA

Within the seven GBD super regions, the second-highest CHD age-standardized death rate (ASDR) was recorded in the MENA. In 2019, CHD was the MENA’s leading cause of death overall, accounting for a quarter of all-cause deaths (799,484.4 deaths) (Table 1), corresponding to an increase of 2% in the CHD death rate (128.9 to 131.3 deaths per 100,000) between 1990 and 2019. However, CHD ASDR decreased by 29% (309.3 to 219 per 100,000) between 1990 and 2019 [6]. At a national level, the three countries with the highest ASDR due to CHD were the Syrian Arab Republic (359.7 [UI 288.3–449.8] per 100,000), Egypt (359.3 [UI 281.8–447.03] per 100,000), and Oman (329.9 [UI 296–364.1] per 100,000), while the three countries with the lowest ASDR were Kuwait (108.5 [UI 90.7–129.2] per 100,000), Turkey (121 [UI 98–147.2] per 100,000), and Jordan (121.9 [UI 103.2–144.1] per 100,000) [6]. Figure 2C shows the variation in CHD ASDR across the MENA [7].

**Fig. 3:**
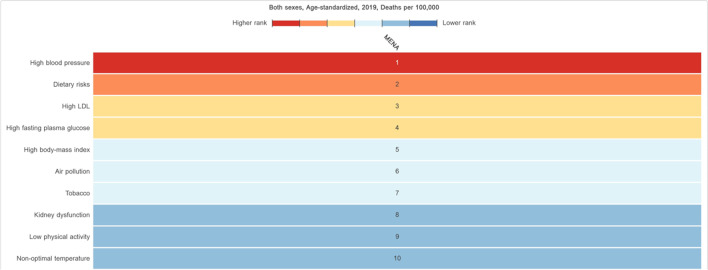
Risk factors with the highest attributable CHD age-standardized death rates in 2019. (Source: The Global Burden of Disease Compare Tool, with permission from: Institute for Health Metrics and Evaluation [7])

**Fig. 4:**
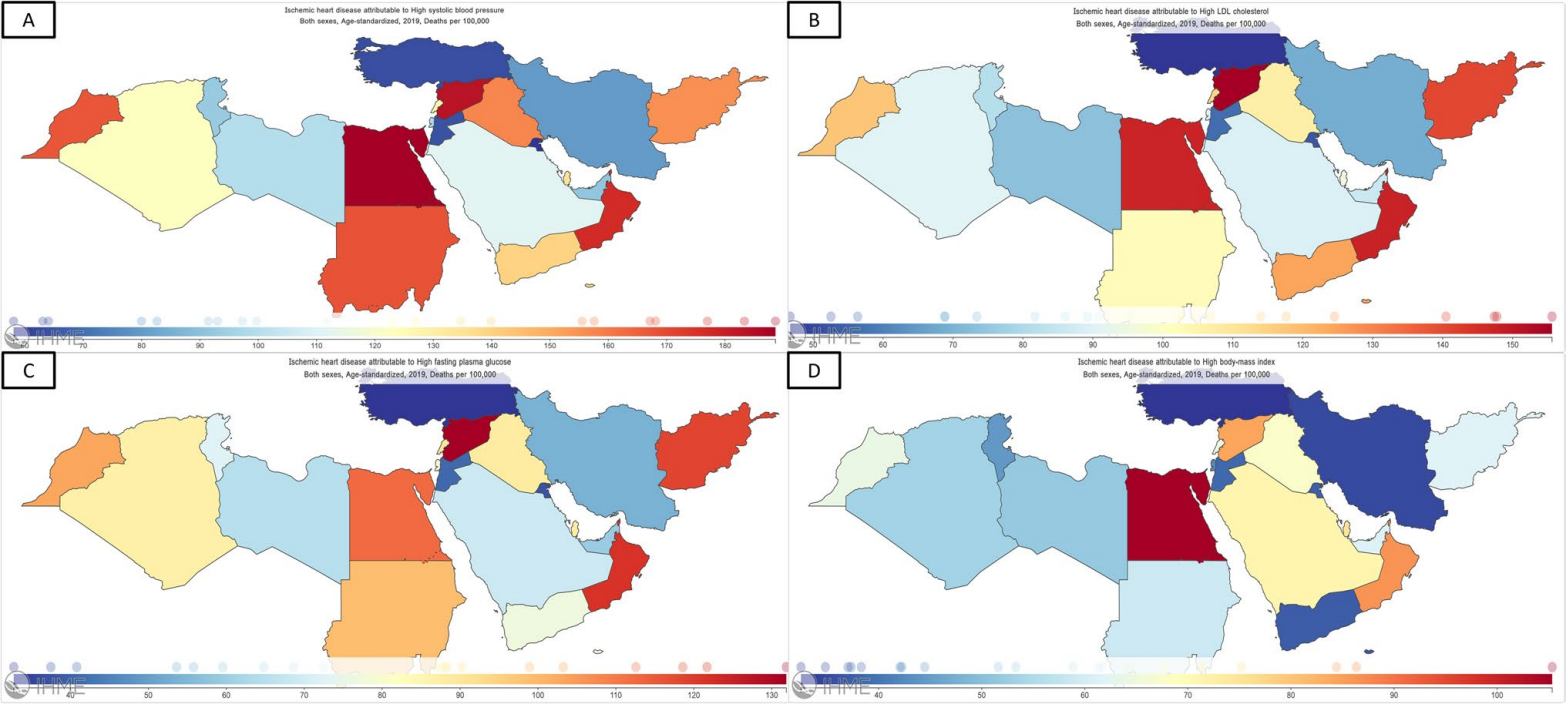
Variations in high systolic blood pressure (**A**), high LDL cholesterol (**B**), high fasting plasma glucose (**C**), and high body mass index (**D**) attributable CHD age-standardized death rates in the MENA in 2019. (Source: The Global Burden of Disease Compare Tool, with permission from: Institute for Health Metrics and Evaluation [7])

Based on data of the GBD 2019 study on level 2 risk factors for CHD death, we highlighted risk factors with the highest attributable burden of CHD ASDR. Including metabolic (high systolic blood pressure, high low-density lipoproteins (LDL) cholesterol, high fasting plasma glucose, high body-mass index (BMI), and kidney dysfunction), behavioral (dietary risks, tobacco, and low physical activity), and environmental (air pollution and non-optimal temperature) risk factors (Fig. 3) [7]. A recent analysis of the GBD 2019 study featured a downward trend in the burden attributed to high systolic blood pressure and high LDL, while the burden of high fasting plasma glucose and high BMI has increased between 1990 and 2019 in the region. Malekpour et al. highlighted as well that the exposure to these cardiometabolic risk factors increased in the past 30 years in the MENA [8]. In this review, we will elaborate more on metabolic risk factors due to their high burden in MENA. Figure 4 shows the variation in metabolic risk factors attributable CHD ASDR in the MENA [7].

### Disability-adjusted Life Years due to CHD in the MENA

Within the seven GBD super regions, the second-highest age-standardized rate of CHD DALY was recorded in the MENA [6]. In 2019, CHD accounted for 11% of the all-cause DALY in the MENA. In 2019, the number of DALY due to CHD was 17,994,821.9 (UI 15,580,582–20,811,862.2) years, corresponding to a decrease of 6% in the rate of DALY (3,148.1 to 2,956.2 years per 100,000), and a reduction of 33% (6,232.4 to 4,158.9 years per 100,00) in the age-standardized rate of DALY between 1990 and 2019. At a national level, the three countries with the highest age-standardized DALY rates were Egypt (6,986.4 [UI 5,361.9–8,973.9] per 100,000), Afghanistan (6,883.5 [UI 5,387.2–8,475.4] per 100,000), and Syrian Arab Republic (6,479.8 [UI 5,077.4–8,364.9] per 100,000), while the three countries with the lowest age-standardized DALY rates were Turkey (2,128.4 [UI 1,728.7–2,583.5] per 100,000), Kuwait (2,252.2 [UI 1,881.6–2,687.8] per 100,000), and Jordan (2,265.2 [UI 1,931.9–2,687.2] per 100,000). Figure 2D shows the variation in CHD age-standardized DALY across the MENA [7].

## Age and Gender Regional Unique Characteristics of Patients with CHD

The INTERHEART study reported that the MENA population presenting with first myocardial infarction (MI) was at least 10 years younger than its Western counterparts, and that the proportion of individuals aged ≤40 years presenting with MI in the MENA was 3-fold higher than in North America and Western Europe [9, 10]. Furthermore, when comparing risk factors in younger vs. older patients presenting with MI/acute coronary syndrome (ACS) in the MENA, younger patients were more likely smokers but had a lower prevalence of metabolic risk factors [9, 11]. However, the burden of these risk factors among the general young patient populations is considerably high in the MENA [12, 13].  In a recent study of 5167 participants from the UAE (mean age of 25.7 years), the age-adjusted prevalence rates for obesity, dysglycemia, dyslipidemia, hypertension, and central obesity were 26.5%, 11.7%, 62.7%, 22.4%, and 22.5%, respectively [12]. Gulf PREVENT, an ongoing case–control study in the United Arab Emirates, will quantify the relative contribution and population-attributable risk percentage associated with premature MI, emphasizing diabetes, obesity, and familial hypercholesterolemia [9].

Women with CHD in the MENA have a higher burden of CVD risk factors, and worse clinical outcomes, and are less likely to receive guideline-recommended therapies and interventions than men [9, 14–18]. In an analysis of 3,224 patients with ACS in Egypt, women had a higher prevalence of hypertension, dyslipidemia, diabetes mellitus (DM), and obesity, while men were more likely smokers and had a higher prevalence of abdominal obesity [15]. El-Menyar et al. reported in their analysis of 8,169 patients with ACS from 6 MENA countries that women were more likely to have CVD risk factors, and more likely to present with unstable angina and were significantly less treated with beta-blockers and antiplatelet therapy [14]. Compared to men, women presenting with ST-segment elevation MI (STEMI) were 90% more likely to die (age-adjusted OR 1.91, 95% CI (1.35–2.66), *P*=0.001) in the MENA [14]. In the Arabian Gulf, Shehab et al. found in their study of 31,620 patients with STEMI that younger women (aged ≤65 years) hospitalized for STEMI were less likely to undergo thrombolysis or primary percutaneous intervention (pPCI) and were less likely to be discharged on guideline-recommended pharmacotherapy. In addition, younger women had higher rates of in-hospital and 1-year mortality than younger men [16].

## Public Awareness and Behavior Toward CHD and its Associated Risk Factors in the MENA

Population-based surveys from the MENA revealed limited public knowledge and awareness of CHD and its associated risk factors [19–23]. Only one in five Emirati women was aware of CHD, and half of the respondents identified left chest pain and dyspnoea as symptoms of CHD [22]. In a survey from Oman, 60.5% of the participants had inadequate CHD knowledge [20]. In Saudi Arabia, respondents were scored according to their awareness of CHD risk factors on a scale of 0 to 14. The mean awareness score was 4.3±1.4, and respondents were mostly aware of fast food and soft drink intake as risk factors for CHD (75% and 64%, respectively). Awareness of other comorbidities among participants was relatively low, such as DM (12%), smoking (26.1%), history of MI (1.5%), and history of stroke (<1%) [23]. In an analysis of healthcare-seeking behavior among Jordanian patients presenting with ACS, delay in seeking care was significantly correlated with patients’ knowledge, attitudes, beliefs, and perception of health [24]. Another major challenge is low adherence to medication among patients with CVD in the MENA; Alomari et al. reported that only half of the patients in the region were adherent to their CVD medications and highlighted that this could be due to specific cultural and personal beliefs that may result in non-adherence [25].

## Current Status of CHD Prevention in the MENA

### Gaps in CHD Prevention in the MENA

Albeit the high burden of CVD risk factors in the region, up to 90% of healthcare expenditure in developed countries is allocated to secondary prevention, while only 5% is allocated to primary care interventions [26, 27]. The WHO service coverage index, which measures coverage of selected essential health services on a scale of 0–100, ranged between 37 and 79 in the MENA in 2019. Turkey, UAE, and Iran had the highest coverage indices, while countries with the lowest indices were Afghanistan, Sudan, and Yemen (Table 1) [28]. However, even in countries with high coverage index, data from the region showed that only half of the patients with chronic conditions had access to medication [29, 30]. In most MENA countries, comprehensive preventive programs with suitable surveillance and monitoring policies for metabolic health are not widely implemented in primary health settings [31]. There is also a lack of national task forces and guidelines addressing non-communicable diseases in the MENA [3]. Additionally, CVD risk assessment tools are still underutilized in the region. Surveys from the MENA revealed that only 7–23% of primary care and family physicians routinely use cardiovascular risk assessment tools despite being aware of these scores [3, 32, 33].

An analysis from the UAE showed poor agreement between six CVD risk tools which considerably impacted decision-making and public health interventions concerning the primary prevention of CVD in the country [34]. In another study from Iran, treatment recommendations were controversial despite a high correlation between risk tools [35]. Furthermore, CVD secondary prevention remains suboptimal in the region [36, 37]. Rabizadeh et al. reported in their study of 323 Iranian patients with DM and CHD that only 7.7% achieved their target goals for blood pressure, LDL, and HbA1c [36].

### Status of Cardiac Rehabilitation in the MENA

Cardiac rehabilitation (CR) has proven to be an effective secondary preventive model of care for CHD, with an associated reduction in CVD morbidity and mortality [38–40]. According to a global survey conducted between 2016 and 2017, the MENA region had 56 CR centers, and around three in five countries had at least one CR center. In MENA countries where CR centers were available, total CR capacities measured 22,181 cases, with a median capacity of 246 (Interquartile range [171-400]) per nation [38]. In the same survey, authors highlighted that lack of financial resources and lack of referrals were the main barrier to greater CR delivery [38, 41]. In another survey that included nine countries (8 of them were from the MENA), only one CR spot was available for every 104 incident CHD cases [41]. This study has shown that CR programs accepted 80% of guideline-indicated patients, but access to these programs takes longer than in other regions of the world. Also, referred patients had to cover the costs partially/totally out of pocket at most of these programs, highlighting a major financial barrier [41]. Moreover, studies have identified the lack of human resources and space as the main barriers to optimal CR care in Arab countries [42]. In addition, women referred to CR prefer women-only classes; such programs were commonly offered in various countries in the region, which is mostly attributable to cultural and religious values in the MENA and might result in greater adherence and psychosocial well-being [43]. There is a need for health policymakers and professional cardiac societies to improve the current referral strategies, facilitate insurance reimbursements, increase the staff-to-patient ratio, and educate healthcare professionals and patients on the importance of CR [38, 44]. Offering CR in an alternate setting (home CR) has been proven to be as effective as center CR in some countries in the region, and broader adaptation might improve adherence to CR and program capacity [44, 45].

## Addressing Metabolic Risk Factors of CHD in the MENA

### Hypertension in the MENA: High Burden, Low Awareness, and Poor Control

High blood pressure is a major modifiable risk factor for CHD [46]. In a recent meta-analysis of 83 studies from the region, the authors found that the overall prevalence of hypertension was 26.2% (95% CI: 24.6–27.9) in the region [47••]. High blood pressure accounted for a quarter of all deaths in the MENA region [8, 31]. Furthermore, more than half of CHD deaths were attributed to high systolic blood pressure (57.3%) in 2019, contributing the most to CHD mortality among all risk factors (Fig. 3) [6, 7]. CHD ASDR due to high systolic blood pressure measured at 117 deaths per 100,000 cases, with the highest burden being recorded in Egypt, followed by the Syrian Arab Republic and Oman (Fig. 4A) [6, 7]. Despite the high burden of hypertension in the MENA region, patients have low awareness and are undertreated [47]. It is estimated that 51.3% (95% CI: 47.7, 54.8) of patients with hypertension were aware of their disease, with half of the patients being treated (47.0% (95% CI: 34.8, 59.2)) and only 43.1% (95% CI: 38.3, 47.9) having their disease control upon treatment [47]. Identified challenges to controlling high blood pressure in the region included the lack of knowledge regarding the importance of screening for hypertension, availability and affordability of medications, patient non-adherence, poor health literacy, and social stigma [48, 49].

### Dyslipidemia: a Highly Prevalent Undertreated Risk Factor for CHD in the MENA

High level of low-density atherogenic lipoproteins is a major risk factor for CHD, and lowering their levels is key to reducing CVD morbidity and mortality [50, 51]. The prevalence of dyslipidemia in the MENA region is high, with a wide range of variation between different study settings and definitions. Epidemiological data from the general population in the region featured a prevalence that varied between 19.2% (Egypt), 32.1 % (Saudi Arabia), and 80.1% (Iran) [52–54]. In the outpatient setting, the Africa Middle East Cardiovascular Epidemiological (ACE) study included 14 countries from the MENA and revealed a prevalence of dyslipidemia of 70% among adult outpatients attending primary care clinics [55]. Recently published outcomes of the PACT-MEA study have shown a dyslipidemia prevalence of 92% in patients with type 2 DM [56]. In the acute setting, around one-third of the patients presenting with ACS were found to have dyslipidemia [57, 58]. In the GBD 2019 study, CHD deaths were attributed to high LDL-C in 46.8% of the cases, and high LDL-C attributable CHD ASDR measured at 91.1 per 100,000 cases, with the highest burden in Syria Arab Republic, Oman, and Egypt (Fig. 4B) [6]. Evidence has shown a correlation between the degree of lowering LDL-C and the reduction in rates of CV events [50]. Compared with less intensive regimens, intensive statin therapy resulted in further reductions in coronary death or non-fatal myocardial infarction of 13% (95% CI 7–19; *P*<0·0001) [50]. Observational studies suggest that achieving LDL-C targets is suboptimal in countries outside of Western Europe [51]. According to various studies and registries from the MENA, one-third to half of treated patients achieve LDL-C targets, with considerable variation among CVD risk groups (Table 2) [51, 53, 59–66]. In a recently published study from Iran, only 43.4% of patients achieved their LDL-C target, which varied across the CVD risk groups. Only one-third of very high-risk patients and two-thirds of the high and moderate-risk groups achieved therapeutic targets [66]. Table 2 shows rates of achieving LDL-C therapeutic targets in the MENA.

**Table 2:** Rates of achieving LDL-C therapeutic targets in the MENA

Study	Publication date	Number of patients, country	Inclusion criteria	CVD risk stratification	Achieving LDL-C target/ hypercholesterolemia control
DYSIS-Egypt[60]^*^	2013	1466, Egypt	Age ≥45 years, and treated for ≥ 3 months with statins	Very high: 85% High: 2.9% Moderate: 8.2% Low: 3.8%	Very high and high (both): 28% Moderate: 50% Overall: 32.8%%
Saudi MOH Survey [61]^#^	2014	10,735, Saudi Arabia	Age≥ 15	–	28.3%#
DYSIS [59]*	2014	2,182, four MENA countries	Age ≥45 years, and treated for ≥ 3 months with statins	Very high: 82.6% High: 2.7% Moderate: 10.3% Low: 4.4%	Very high: 30.5% High: 43.1% Moderate: 71% Low: 100% Overall: 38.2%
CEPHEUS [62]^$^	2014	5276, Arabian Gulf Countries	Age≥ 18, taking LLT for 3 months,	Very high: 32.9% High: 44.3% Medium-high:3.3% Medium-low:7.5% Low: 12.1%	Very high: 31.9% High: 52.7% Medium-high:33% Medium-low: 81% Low: 91% Overall: 52%
CEPHEUS I Egypt [63]^$^	2014	1034, Egypt	Age≥ 18, taking LLT for 3 months	Very high: 37.7% High: 34.7% Medium-high: 3.6% Medium-low: 16.0% Low: 8%	Very high: 10.7% High: 34.2% Medium-high:7.9% Medium-low: 66.5% Low: 71.1% Overall: 32.5%
CEPHEUS II Egypt [64]*	2017	1127, Egypt	Age≥ 18, taking LLT for 3 months,	Very high: 65.2% High: 2.5% Moderate: 20% Low: 12.4%	Very high: 22.3% High: 18.2% Moderate: 44.7% Low: 84.7% Overall: 34.4%
DYSIS II—Egypt [65]*	2018	199, Egypt	Age≥ 18, hospitalized for ACS	–	Very High: 5.1% High: 27.3% Moderate: 32.3% Low: 14.3% Overall: –
ICLPS [51]*	2018	9049, 26.2% of them were from the MENA	Treated for ≥ 3 months with any LLT	Very high: 60.9% High: 33% Moderate: 5.2% Low: 0.9%	Very high: 32.1% High: 51.9% Moderate: 55.7% Low: – Overall: 39.9%
Aghasizadeh et al. [66]^+^	2021	576, Iran	Treated with a statin for 12 months	Extreme: 1.6% Very high: 58.5% High: 9.5% Moderate: 30.4%	Extreme: 0% Very high: 28.2% High: 70.9% Moderate: 64.6% Overall: 42.9%
PACT-MEA [56] ++	2023	3726, 6/7 countries were from the MENA	Age≥ 18 years were diagnosed with T2D ≥ 180 days prior to study	Very high: 29.9% High: 69.4%%	High/very high: 30%

*ACS* acute coronary syndrome, *CEPHEUS* centralized pan-Middle East survey on the undertreatment of hypercholesterolemia, *CVD* cardiovascular disease, *DYSIS* Dyslipidemia International Study, *ICLPS* International Cholesterol management Practice Study, *LDL-C* low-density lipoprotein-cholesterol, *LLT* lipid-lowering therapy, *MENA* Middle East and North Africa, *MOH* Ministry of Health

*The 2011 version of the European Society of Cardiology (ESC) guidelines was used to define cardiovascular risk and abnormalities in LDL-C and patients were risk-stratified according to the Systematic Coronary Risk Estimation (SCORE) Chart

#Hypercholesterolemia was considered controlled if measured cholesterol levels were below 6.2 mmol/L

$Patient risk categorization, respective LDL-C goals, and target attainment were determined according to the 2004 updated National Cholesterol Education Program Adult Treatment Panel III (NCEP ATP III)

+Atherosclerotic cardiovascular diseases risk level and treatment goals were determined according to the American Association of Clinical Endocrinologists (AACE) 2017 guidelines

++Cardiovascular risk categories were determined according to the European Society of Cardiology [ESC] 2021 guidelines, with an LDL-C target of<70 mg/dL

Additionally, there is limited physician awareness of target LDL-C among patients with CHD in the MENA and limited optimization of other lipid therapeutic goals such as non-high-density lipoprotein-cholesterol (HDL-C) and ApoB [67, 68]. The 2021 updated clinical recommendations for the management of lipid disorders in the Middle East were established using updated international guidelines and epidemiological evidence from the Gulf region. These recommendations highlighted the need for more intensive reductions of LDL-C and to consider non-HDL-C as a primary treatment target in the region. Lifestyle modifications and statins remain the first-line treatment recommendation and the first-line pharmacological therapy for all patients, respectively [69].

### Diabetes Mellitus: a High Prevalence of CHD and the Need to Adapt Novel Cardioprotective Therapies in the MENA

The risk of CHD is two to six-fold higher in patients with type 2 DM than in those without DM [70]. In 2021, a sixth of the population in the MENA region had type 2 DM, which is considered the highest regional prevalence [71]. In the MENA, CHD deaths were attributed to high fasting blood glucose in one-third of the cases, and CHD ASDR due to high fasting blood glucose was estimated at 72.5 per 100,000 cases, with the highest burden being recorded in the Syrian Arab Republic, followed by Oman and Afghanistan (Fig. 4C) [6, 7].

Recently published multi-center studies investigated the prevalence of CHD among type 2 DM patients in the MENA [56, 72, 73]. The DISCOVER study included ten countries from the MENA region, and estimated that 12.7% of patients initiated on second-line treatment for type 2 DM had macrovascular complications (including CHD) [72]. The CAPTURE study, which included multiple countries from the MENA, estimated an overall weighted prevalence of CHD of 17.7% in patients with type 2 DM [73]. Similarly, Verma et al. reported that one-fifth of patients with type 2 DM in the Middle East had an established atherosclerotic CVD (ASCVD), and CHD accounted for most of these cases. The authors also reported that the highest CHD burden in patients with DM was in Bahrain and the UAE [56]. In a recent study from Iran of patients undergoing coronary artery bypass surgery, DM was significantly associated with developing triple vessel CHD and requiring > three grafts during the surgery [74]. However, DM did not predict in-hospital major adverse events among the population [74]. In patients undergoing coronary revascularization in Jordan, patients with DM were more likely to require PCI during follow-up [75].

Major professional diabetes and cardiac associations have recommended glucose-lowering agents with CV benefits, including sodium-glucose co-transporter-2 (SGLT2) inhibitors and glucagon-like peptide-1 (GLP-1) agonists, to patients with type 2 DM and established/at risk of ASCVD [76]. In the MENA region, the rate of utilizing cardioprotective lowering medications ranged between 8.3 and 13% for GLP-1 agonists and 15.1 and 37% for SGLT2 inhibitors among eligible CVD patients with type 2 DM enrolled in the PACT-MEA and CAPTURE studies [56, 73, 77].

### Unraveling the Impact of Obesity, Physical Inactivity, and Dietary Patterns on CHD Risk

The prevalence of obesity in the MENA is high (21.17%), and about 30.3% of CHD deaths were attributed to high BMI in 2019 [6, 78]. CHD ASDR due to high BMI measured at 56 deaths per 100,000 cases in the region, with the highest burden being recorded in Egypt, followed by Oman and the Syrian Arab Republic (Fig. 4D) [7]. Moreover, in a recent meta-analysis from the MENA, 49.2% of adults (ranging from 5.1% in Jordan to 86.8% in Sudan) and 74.4% of youth (ranging from 49% in Lebanon to 91.7% in Egypt) were not sufficiently physically active [79]. In a recent survey from the MENA, it was found that three in four patients with CHD were physically inactive, and the authors also identified barriers to physical activity, among others, anxiety, lack of interest, and lack of time [80]. In those with CHD and inadequate activity, evidence from the MENA supports the efficacy of multifaceted behavioral interventions in increasing physical activity and improving physiological and psychological outcomes [81]. Dietary risks have been found to have the second highest attributable burden on CHD mortality in the MENA [7]. MENA countries have undergone a nutritional shift, transitioning from diets rich in fruits, vegetables, and whole grains toward a Westernized diet rich in meats, fatty foods, and sweets [82, 83]. In the Prospective Urban Rural Epidemiology (PURE) study, participants from Middle East countries consumed more than 30% and 10% of their energy from fat and saturated fatty acids, respectively [84]. This aligns with the findings of a recent survey from the UAE, revealing that almost two-thirds of the participants frequently/consistently considered eating fried food as their main course [85]. Aljefree et al. reported a strong association between the Western diet and CHD in the MENA [86]. Also, Mohammadifard et al. reported that high adherence to Mediterranean dietary patterns was found to reduce CV mortality by 46% [87]. Furthermore, legume intake was inversely correlated with CVD risk, according to a 7-year follow-up study from Iran [88].

### Smoking: a Major Modifiable Risk Factor for CHD in the MENA

The prevalence of smoking varies in the region ranging from as low as 20% (Bahrain) to 35% (Lebanon) [89, 90]. Among patients with ACS enrolled in the GULF RACE registry, 38% were smokers, and cigarette smokers tended to have typical and earlier presentations than non-smokers [91]. Amiri et al. reported in their 12-year follow-up study of 10,400 CVD-free participants in Iran that in comparison to never smokers, the adjusted hazard ratios of CHD were 1.2, 2, and 2.1 in past smokers, occasional smokers, and daily smokers, respectively [92]. In addition, quitters for ≥15 years were almost CHD risk-free [92].

## Suboptimal Management of ACS in the MENA

### Underutilization of Emergency Medical Services in the Setting of ACS

In patients with ACS, the time from symptom onset to the initiation of reperfusion therapy is a key determinant of prognosis and myocardial salvage [93]. Major professional cardiac societies have recommended activation of the emergency medical service (EMS) by symptomatic patients suspected to have ACS, which facilitates the process of care and decision-making and reduces morbidity and mortality [93, 94]. In the MENA, studies have shown that only 17 to 28% of patients presenting with ACS were transported by EMS [95–97]. Shehab et al. found that both groups of patients who received timely vs. delayed PCI had low ambulance service use rates (27.7% vs. 16.6%; *P*=.06) [96]. In a multi-center analysis from 6 MENA countries of patients presenting to the emergency department (ED) with ACS, only 17% were transported using EMS. Additionally, EMS-transported patients presenting with STEMI were found to present earlier, had shorter door-to-electrocardiogram time, and were more likely to receive reperfusion therapy within 30 min of arrival at the ED [95]. ESC guidelines recommend conducting a resting 12-lead ECG at first contact with EMS in the pre-hospital setting and its immediate interpretation by a qualified physician [98]. However, data show low adaptation of this recommendation. In a prospective registry of 36 hospitals in 6 Arabian Gulf countries, most ECGs were performed in clinics or non-PCI hospitals rather than in the ambulance. Additionally, the study reported that a significant proportion of ambulance paramedics lacked BLS and ACLS certifications [99].

### Limited Availability of pPCI for Patients with STEMI in the MENA

Timely pPCI is the gold-standard reperfusion therapy for patients presenting with STEMI [100]. In the MENA region, published studies between 2008 and 2014 reported a rate of 7–40% of pPCI among patients presenting with STEMI [96, 101–103]. More recent studies from Egypt and the Gulf region have shown an increase in rates of pPCI, achieving 56.4% (Table 3) [99, 104–106].

**Table 3:** Accessibility to primary PCI in patients presenting with STEMI in the MENA

Study	Publication year, country	Number of STEMI patients	Rate of pPCI%
Gulf RACE [103]	2008, Gulf Area	549	7%
ACCESS Registry [102]	2011, 28% of patients were from the Middle East	5411	40%
SPACE Registry [101]	2011, Saudi Arabia	2096	17.5%
Gulf RACE II [96]	2014, Gulf region	3432	11%
Gulf RACE-3Ps [99]	2016, Gulf region	2928	46%
STARS-1 Program [105]	2019, Saudi Arabia	1471	42.5%
Shaheen et al. [106]	2020, Egypt	1356	49.1%
PEACE MENA Registry [104]	2021, MENA	312	56.4%

*Gulf RACE* Gulf Registry of Acute Coronary Events, *PEACE MENA* Program for the Evaluation and Management of the Cardiac Events Registry for the MENA region, *SPACE* Saudi Project for Assessment of Coronary Events, *STARS-1 Program* Saudi Acute Myocardial Infarction Registry Program, *STEMI* ST-elevation Myocardial Infarction

In a recently published analysis of the PEACE MENA registry that included 14 Arab countries from the MENA, only 56.4% of patients presenting with STEMI received pPCI, while 24% received thrombolysis, and 19.5% did not receive reperfusion therapy [104]. Furthermore, compared to STEMI patients with higher incomes, those with lower incomes were less likely to receive timely pPCI [104]. Major identified challenges toward the implementation of pPCI in the management of patients presenting with STEMI included late presentation due to delays in the pre-hospital and emergency department care, prior thrombolysis, unavailability of trained operators and equipment, limited number of 24/7 cath labs, limited public medical insurance coverage, and lack of STEMI networks and hospital policies [106–108]. Recent data from the region have shown that the utilization of high-sensitivity cardiac troponin T assays in the emergency department increased MI diagnosis by 23%; wider adoption of these assays in the region could help inform decision-making and resource utilization in patients with ACS [109]. In developed countries, regional networks among primary PCI-capable and non-capable hospitals play a key role in managing patients with STEMI [110]. A recently published study on implementing such networks in Egypt has proven its feasibility and efficacy with increased rates of pPCI, better clinical outcomes, and more optimal hospital resource utilization [110]. Table 3 shows rates of access to primary PCI in patients presenting with STEMI in the MENA.

## Future Directions to Mitigate the Pandemic of CHD in the MENA

Considering the high prevalence of cardiometabolic risk factors in the region, patients may benefit from a multi-disciplinary cardiometabolic clinic model of care where cardio-preventive and weight-loss medications, behavioral counseling, and diet and lifestyle interventions are all provided at once [77]. All stakeholders involved in healthcare should work together to develop and execute strategies to tackle the burden of CHD in the MENA. Such strategies should prioritize incorporating universal health coverage in the region; organizing health campaigns aimed at increasing public awareness of CHD and its risk factors; adapting effective strategies for screening, diagnosing, treating, and monitoring CHD risk factors (including dyslipidemia, hypertension, etc.); and emphasizing patient education about CHD implications, adherence to medications, lifestyle modifications, and follow-up appointments [30, 47••, 111]. Furthermore, there is a need for clinical research initiatives in the region, including multi-center clinical trials, to pave the way to develop regional standardized definitions, risk tools, and guidelines [67]. Finally, efforts should be made to better adhere to guideline-recommended therapies in women with CHD and recruit more women from the region into clinical trials to improve CHD awareness, prevention, detection, and treatment [112].

## Conclusion

CHD is the leading cause of morbidity, mortality, and disability in the Middle East and North Africa. However, despite the high burden of CHD risk factors, many of these factors remain underrecognized, undertreated, and uncontrolled. Additionally, CHD care is hampered by poor patient awareness, inefficient preventive strategies, and limited access to guideline-recommended therapeutics. Therefore, there is a need for all stakeholders involved in healthcare to work together to develop and execute strategies aimed at tackling the burden of CHD in the MENA.

## Abbreviations

ACS: Acute coronary syndrome
ASCVD: Atherosclerotic cardiovascular disease
ASDR: Age-standardized death rate
BMI: Body mass index
CHD: Coronary heart disease
CR: Cardiac rehabilitation
CVD: Cardiovascular disease
DALY: Disability-adjusted life years
DM: Diabetes mellitus
EMS: Emergency medical services
GBD: Global burden of disease
GLP-1: Glucagon-like peptide-1
LDL: Low-density lipoprotein
MENA: Middle East and North Africa
MI: Myocardial infarction
pPCI: Primary percutaneous coronary intervention
SDI: Socio-demographic index
SGLT2: Sodium-glucose co-transporter-2
STEMI: ST-elevation myocardial infarction
UI: Uncertainty interval

## References

[1] Institute for Health Metrics and Evaluation. Frequently asked questions. Institute for Health Metrics and Evaluation. https://www.healthdata.org/gbd/faq#What%20countries%20are%20in%20each%20region?. Accessed 10 Apr 2023.

[2] Global Burden of Disease Collaborative Network (2020). Global burden of disease study 2019 (GBD 2019) population estimates 1950-2019.

[3] Turk-Adawi K, Sarrafzadegan N, Fadhil I, Taubert K, Sadeghi M, Wenger NK, Tan NS, Grace SL (2018). Cardiovascular disease in the Eastern Mediterranean region: epidemiology and risk factor burden. Nat Rev Cardiol..

[4] Global Burden of Disease Collaborative Network Global burden of disease study 2019 (GBD 2019) socio-demographic index (SDI) 1950–2019. Seattle, United States of America: Institute for Health Metrics and Evaluation (IHME), 2020. 10.6069/D8QB-JK35. Accessed 7 Apr 2023.

[5] Lancet T (2018). GBD 2017: a fragile world. Lancet..

[6] Global Burden of Disease Collaborative Network (2020). Global burden of disease study 2019 (GBD 2019) results.Seattle, United States.

[7] Institute for Health Metrics and Evaluation (IHME). GBD compare. Seattle, WA: IHME. University of Washington; 2015. Available from http://vizhub.healthdata.org/gbd-compare. (Accessed [03/12/2022]))

[8] •• Malekpour M-R, Abbasi-Kangevari M, Ghamari S-H, Khanali J, Heidari-Foroozan M, Moghaddam SS, Azangou-Khyavy M, Rezazadeh-Khadem S, Rezaei N, Shobeiri P. The burden of metabolic risk factors in North Africa and the Middle East, 1990–2019: findings from the Global Burden of Disease study. E Clin Med. 2023;60 **This analysis highlights 30 years of trends in the burden attributable to cardiometabolic risk factors in the region**

[9] Dugani SB, Murad W, Damilig K (2020). Premature myocardial infarction in the Middle East and North Africa: rationale for the Gulf PREVENT study. Angiology..

[10] Yusuf S, Hawken S, Ôunpuu S, Dans T, Avezum A, Lanas F, McQueen M, Budaj A, Pais P, Varigos J (2004). Effect of potentially modifiable risk factors associated with myocardial infarction in 52 countries (the INTERHEART study): case-control study. The lancet..

[11] Obeidat OS, Makhamreh H, Al-Muhaisen RZ (2021). Clinical characteristics and prognosis of young Middle Eastern adults with ST-elevation myocardial infarction: one-year follow-up. Heart Views..

[12] Mezhal F, Oulhaj A, Abdulle A, AlJunaibi A, Alnaeemi A, Ahmad A, Leinberger-Jabari A, Al Dhaheri AS, AlZaabi E, Al-Maskari F (2023). High prevalence of cardiometabolic risk factors amongst young adults in the United Arab Emirates: the UAE Healthy Future study. BMC Cardiovasc Disord..

[13] AlMuhaidib S, AlBuhairan F, Tamimi W, AlDubayee M, AlAqeel A, Babiker A, AlFaraidi H, AlJuraibah F, Badri M, Al Alwan I (2022). Prevalence and factors associated with dyslipidemia among adolescents in Saudi Arabia. Sci Rep..

[14] El-Menyar A, Zubaid M, Rashed W, Almahmeed W, Al-Lawati J, Sulaiman K, Al-Motarreb A, Amin H, Singh R, Al Suwaidi J (2009). Comparison of men and women with acute coronary syndrome in six Middle Eastern countries. Am J Cardiol..

[15] Reda A, Bendary A, Elbahry A, Farag E, Mostafa T, Khamis H, Wadie M, Bendary M, Azeem BA, Salah R (2020). Prevalence of atherosclerosis risk factors in Egyptian patients with acute coronary syndrome: final data of the nationwide cross-sectional ‘CardioRisk’ project. J Public Health Afr..

[16] Shehab A, Bhagavathula AS, Alhabib KF, Ullah A, Suwaidi J, Al AW, AlFaleh H, Zubaid M (2020). Age-related sex differences in clinical presentation, management, and outcomes in ST-segment–elevation myocardial infarction: pooled analysis of 15 532 patients from 7 Arabian Gulf registries. J Am Heart Assoc..

[17] Jarrah MI, Hammoudeh AJ, Al-Natour DB, Khader YS, Tabbalat RA, Alhaddad IA, Kullab SM (2017). Gender differences in risk profile and outcome of Middle Eastern patients undergoing percutaneous coronary intervention. Saudi Med J..

[18] AlQuaiz AM, Kazi A, Alodhayani AA, Almeneessier A, AlHabeeb KM, Siddiqui AR (2021). Age and gender differences in the prevalence of chronic diseases and atherosclerotic cardiovascular disease risk scores in adults in Riyadh City, Saudi Arabia. Saudi Med J..

[19] Awad A, Al-Nafisi H (2014). Public knowledge of cardiovascular disease and its risk factors in Kuwait: a cross-sectional survey. BMC Public Health..

[20] Ammouri AA, Tailakh A, Isac C, Kamanyire JK, Muliira J, Balachandran S (2016). Knowledge of coronary heart disease risk factors among a community sample in Oman: pilot study. Sultan Qaboos Univ Med J..

[21] Mukattash TL, Shara M, Jarab AS, Al-Azzam SI, Almaaytah A, Al Hamarneh YN (2012). Public knowledge and awareness of cardiovascular disease and its risk factors: a cross-sectional study of 1000 Jordanians. Int J Phar Prac..

[22] Khan S, Ali SA (2017). Exploratory study into awareness of heart disease and health care seeking behavior among Emirati women (UAE)-cross sectional descriptive study. BMC Womens Health..

[23] Almalki MA, AlJishi MN, Khayat MA, Bokhari HF, Subki AH, Alzahrani AM, Alhejily WA. Population awareness of coronary artery disease risk factors in Jeddah Saudi Arabia: a cross-sectional study. Int J Gen Med. 2019:63–70. 10.2147/IJGM.S184732 PMC6333320 30666149

[24] Darawad MW, Alfasfos N, Saleh Z, Saleh AM, Hamdan-Mansour A (2016). Predictors of delay in seeking treatment by Jordanian patients with acute coronary syndrome. Int Emerg Nurs..

[25] Alomari A, Alananzeh I, Lord H, Abdulla Al-Lenjawi B, Fernandez R (2022). Medication adherence rate in Arab Patients With cardiovascular disease: a systematic review. J Trans Nurs..

[26] Bhagavathula AS, Shehab A, Ullah A, Rahmani J (2020). The burden of cardiovascular disease risk factors in the Middle East: a systematic review and meta-analysis focusing on primary prevention. Curr Vasc Pharmacol..

[27] Alasnag M, Awan Z, Al Ghamdi A, Al Modaimeigh H, Al Shemiri M (2020). Improvement initiative in LDL-C management in Saudi Arabia: a call to action. IJC Heart & Vasculature..

[28] GHO (2023). By category Index of service coverage - data by country.

[29] Vialle-Valentin CE, Serumaga B, Wagner AK, Ross-Degnan D (2015). Evidence on access to medicines for chronic diseases from household surveys in five low-and middle-income countries. Health Policy Plan..

[30] Abboud M, Karam S (2022). Hypertension in the Middle East: current state, human factors, and barriers to control. J Hum Hypertens..

[31] Azizi F, Hadaegh F, Hosseinpanah F (2019). Metabolic health in the Middle East and north Africa. Lancet Diabetes Endoc..

[32] Ahmed AA, Alsharief E, Alsharief A (2013). Evaluation of risk factors for cardiovascular diseases among Saudi diabetic patients attending primary health care service Diabetes & Metabolic Syndrome. Clin Res Rev..

[33] Nour-Eldein H, Abdelsalam SA, Nasr GM, Abdelwahed HA (2013). Global cardiovascular risk assessment by family physicians in Suez canal university-family medicine centers-Egypt. J Family Med Prim Care..

[34] Oulhaj A, Bakir S, Aziz F, Suliman A, Almahmeed W, Sourij H, Shehab A (2020). Agreement between cardiovascular disease risk assessment tools: an application to the United Arab Emirates population. PLoS One..

[35] Motamed N, Rabiee B, Perumal D, Poustchi H, Miresmail SJH, Farahani B, Maadi M, Saeedian FS, Ajdarkosh H, Khonsari MR (2017). Comparison of cardiovascular risk assessment tools and their guidelines in evaluation of 10-year CVD risk and preventive recommendations: a population based study. Int J Cardiol..

[36] Rabizadeh S, Mansournia MA, Salehi SS, Khaloo P, Alemi H, Mirbolouk H, Blaha MJ, Esteghamati A, Nakhjavani M (2019). Comparison of primary versus secondary prevention of cardiovascular disease in patients with type2 diabetes: focus on achievement of ABC goals Diabetes & Metabolic Syndrome. Clin Res Rev..

[37] Yusuf S, Islam S, Chow CK, Rangarajan S, Dagenais G, Diaz R, Gupta R, Kelishadi R, Iqbal R, Avezum A (2011). Use of secondary prevention drugs for cardiovascular disease in the community in high-income, middle-income, and low-income countries (the PURE study): a prospective epidemiological survey. The Lancet..

[38] Turk-Adawi K, Supervia M, Lopez-Jimenez F (2019). Cardiac rehabilitation availability and density around the globe. E Clin Med..

[39] Anderson L, Thompson DR, Oldridge N, Zwisler A, Rees K, Martin N, Taylor RS. Exercise-based cardiac rehabilitation for coronary heart disease. Cochrane Database of Sys Rev. 2016; 10.1002/14651858.CD001800.pub3 PMC6491180 26730878

[40] Shields GE, Wells A, Doherty P, Heagerty A, Buck D, Davies LM (2018). Cost-effectiveness of cardiac rehabilitation: a systematic review. Heart..

[41] Turk-Adawi K, Supervia M, Pesah E (2019). Availability and delivery of cardiac rehabilitation in the Eastern Mediterranean Region: how does it compare globally?. Int J Cardiol..

[42] Turk-Adawi KI, Terzic C, Bjarnason-Wehrens B, Grace SL (2015). Cardiac rehabilitation in Canada and Arab countries: comparing availability and program characteristics. BMC Health Serv Res..

[43] Turk-Adawi K, Supervia M, Lopez-Jimenez F, Adawi A, Sadeghi M, Grace SL (2021). Women-only cardiac rehabilitation delivery around the world. Heart Lung Circ..

[44] Ragupathi L, Stribling J, Yakunina Y, Fuster V, McLaughlin MA, Vedanthan R (2017) Availability, use, and barriers to cardiac rehabilitation in LMIC. Glob Heart 12:323-334.e10 10.1016/j.gheart.2016.09.004 28302548

[45] Dalal HM, Zawada A, Jolly K, Moxham T, Taylor RS. Home based versus centre based cardiac rehabilitation. Cochrane systematic review and meta-analysis. Bmj. 2010:340. 10.1136/bmj.b5631 PMC2808470 20085991

[46] Weber T, Lang I, Zweiker R, Horn S, Wenzel RR, Watschinger B, Slany J, Eber B, Roithinger FX, Metzler B (2016). Hypertension and coronary artery disease: epidemiology, physiology, effects of treatment, and recommendations: a joint scientific statement from the Austrian Society of Cardiology and the Austrian Society of Hypertension. Wien Klin Wochenschr..

[47] Balouchi A, MHAP R, Al-Mutawaa K, Naderifar M, Rafiemanesh H, Ebadi A, Ghezeljeh TN, Shahraki-Mohammadi A, Al-Mawali A (2022). Hypertension and pre-hypertension in Middle East and North Africa (MENA): a meta-analysis of prevalence, awareness, treatment, and control. Curr Probl Cardiol..

[48] Al Qasem A, Smith F (2011). Clifford S (2011) Adherence to medication among chronic patients in Middle Eastern countries: review of studies. EMHJ-Eastern Medit Health J..

[49] Akl C, Akik C, Ghattas H, Obermeyer CM (2020). The cascade of care in managing hypertension in the Arab world: a systematic assessment of the evidence on awareness, treatment and control. BMC Public Health..

[50] Baigent C, Blackwell L, Emberson J (2010). Efficacy and safety of more intensive lowering of LDL cholesterol: a meta-analysis of data from 170 000 participants in 26 randomised trials. The Lancet..

[51] Danchin N, Almahmeed W, Al-Rasadi K, Azuri J, Berrah A, Cuneo CA, Karpov Y, Kaul U, Kayıkçıoğlu M, Mitchenko O (2018). Achievement of low-density lipoprotein cholesterol goals in 18 countries outside Western Europe: the International ChoLesterol management Practice Study (ICLPS). Eur J Prev Cardiol..

[52] Aryan Z, Mahmoudi N, Sheidaei A (2018). The prevalence, awareness, and treatment of lipid abnormalities in Iranian adults: surveillance of risk factors of noncommunicable diseases in Iran 2016. J Clin Lipidol..

[53] Reda A, Ragy H, Saeed K, Alhussaini MA (2021). A semi-systematic review on hypertension and dyslipidemia care in Egypt—highlighting evidence gaps and recommendations for better patient outcomes. J Egyp Public Health Assoc..

[54] Alhabib KF, Batais MA, Almigbal TH, Alshamiri MQ, Altaradi H, Rangarajan S, Yusuf S (2020). Demographic, behavioral, and cardiovascular disease risk factors in the Saudi population: results from the Prospective Urban Rural Epidemiology study (PURE-Saudi). BMC Public Health..

[55] Alsheikh-Ali AA, Omar MI, Raal FJ, Rashed W, Hamoui O, Kane A, Alami M, Abreu P, Mashhoud WM (2014). Cardiovascular risk factor burden in Africa and the Middle East: the Africa Middle East cardiovascular epidemiological (ACE) study. PLoS One..

[56] •• Verma S, Alamuddin N, Alawadi F, Alkandari H, Al Mahmeed W, Assaad-Khalil SH, Haddad J, Husemoen LLN, Lombard L, Malik RA. Prevalence of diabetes and cardiovascular risk in the Middle East and Africa: primary results of the PACT-MEA study. Circulation. 2023; The study provides the first contemporary prevalence estimates of atherosclerotic cardiovascular disease among those with type 2 diabetes mellitus in seven countries from the Middle East and Africa 10.1161/CIRCULATIONAHA.123.064345 PMC10101130 36877670

[57] El-Menyar A, Zubaid M, Shehab A, Bulbanat B, Albustani N, Alenezi F, Al-Motarreb A, Singh R, Asaad N, Al Suwaidi J (2011). Prevalence and impact of cardiovascular risk factors among patients presenting with acute coronary syndrome in the Middle East. Clin Cardiol..

[58] AlHabib KF, Sulaiman K, Al-Motarreb A, Almahmeed W, Asaad N, Amin H, Hersi A, Al-Saif S, AlNemer K, Al-Lawati J (2012). Baseline characteristics, management practices, and long-term outcomes of Middle Eastern patients in the second Gulf Registry of Acute Coronary Events (Gulf RACE-2). Ann Saudi Med..

[59] Al Sifri SN, Almahmeed W, Azar S, Okkeh O, Bramlage P, Jünger C, Halawa I, Ambegaonkar B, Wajih S, Brudi P (2014). Results of the Dyslipidemia International Study (DYSIS)-Middle East: clinical perspective on the prevalence and characteristics of lipid abnormalities in the setting of chronic statin treatment. PLoS One..

[60] El Etriby A, Bramlage P, El Nashar A, Brudi P (2013). The DYSlipidemia International study (DYSIS)-Egypt: A report on the prevalence of lipid abnormalities in Egyptian patients on chronic statin treatment. Egyptian Heart J..

[61] Basulaiman M, El Bcheraoui C, Tuffaha M (2014). Hypercholesterolemia and its associated risk factors—Kingdom of Saudi Arabia, 2013. Ann Epidemiol..

[62] Arafah M, Al-Hinai AT, Mahmeed W Al, Al-Rasadi K, Tamimi O Al, Herz S Al, Anazi F Al, Nemer K Al, Metwally O, Alkhadra A (2014) Centralized pan-Middle East survey on the undertreatment of hypercholesterolemia: results from the CEPHEUS study in Arabian Gulf countries. Angiology. 65:919–926 10.1177/0003319713512414 24301426

[63] Reda A, Abdel-Rehim AA, Etman A, Afifi OSA (2014). Centralized pan-Middle East survey on the under-treatment of hypercholesterolemia: results from the CEPHEUS study in Egypt. Cardiol Ther..

[64] Reda A, Etman A, Abdel-Rahim A, Farag N, Sanad O, Salamah S (2017). Centralized pan-Middle East survey on the under-treatment of hypercholesterolemia: results from the CEPHEUS II study in Egypt. Cardiol Ther..

[65] Sobhy M, El Etriby A, El Nashar A, Wajih S, Horack M, Brudi P, Lautsch D, Ambegaonkar B, Vyas A, Gitt AK (2018). Prevalence of lipid abnormalities and cholesterol target value attainment in Egyptian patients presenting with an acute coronary syndrome. Egyptian Heart J..

[66] Aghasizadeh M, Bizhaem SK, Baniasadi M, Khazdair MR, Kazemi T (2021). Evaluation of LDL goal achievement in statin consumption, south east of Iran. Sci Rep..

[67] Al Rasadi K, Almahmeed W, AlHabib KF (2016). Dyslipidaemia in the Middle East: current status and a call for action. Atherosclerosis..

[68] Al-Omran M. Atherosclerotic disease and risk factor modification in Saudi Arabia: a call to action. Vasc Health Risk Manag. 2012:349–55. 10.2147/VHRM.S32783 PMC3373315 22701328

[69] Alsayed N, Almahmeed W, Alnouri F, Al-Waili K, Sabbour H, Sulaiman K, Zubaid M, Ray KK, Al-Rasadi K (2022). Consensus clinical recommendations for the management of plasma lipid disorders in the Middle East: 2021 update. Atherosclerosis..

[70] Sasso FC, Carbonara O, Nasti R, Campana B, Marfella R, Torella M, Nappi G, Torella R, Cozzolino D (2004). Glucose metabolism and coronary heart disease in patients with normal glucose tolerance. JAMA..

[71] Federation ID (2021). IDF Diabetes Atlas, tenth.

[72] Gomes MB, Rathmann W, Charbonnel B, Khunti K, Kosiborod M, Nicolucci A, Pocock SJ, Shestakova MV, Shimomura I, Tang F (2019). Treatment of type 2 diabetes mellitus worldwide: baseline patient characteristics in the global DISCOVER study. Diabetes Res Clin Pract..

[73] Mosenzon O, Alguwaihes A, Leon JLA, Bayram F, Darmon P, Davis TME, Dieuzeide G, Eriksen KT, Hong T, Kaltoft MS (2021). CAPTURE: a multinational, cross-sectional study of cardiovascular disease prevalence in adults with type 2 diabetes across 13 countries. Cardiovasc Diabetol..

[74] Nomali M, Ayati A, Tayebi A, Heidari ME, Moghaddam K, Mosallami S, Riahinokandeh G, Nomali M, Roshandel G (2023). Type 2 diabetes mellitus and in-hospital major adverse cardiac and cerebrovascular events (MACCEs) and postoperative complications among patients undergoing on-pump isolated coronary artery bypass surgery in Northeastern Iran. BMC Cardiovasc Disord..

[75] Alhaddad IA, Tabbalat R, Khader Y, Elkarmi Z, Dahabreh Z, Hammoudeh A (2021) Surviving a decade or more after coronary revascularization in a middle Eastern population: the impact of diabetes mellitus. 10.4103/HEARTVIEWS.HEARTVIEWS_36_21 PMC9542966 36213429

[76] Verma S, Sabbour H, Alamuddin N, Alawadi F, Alkandari H, Almahmeed W, Assaad-Khalil SH, Haddad J, Lombard L, Malik RA. A cross-sectional study of the prevalence and clinical management of atherosclerotic cardiovascular diseases in patients with type 2 diabetes across the Middle East and Africa (PACT-MEA): study design and rationale. Diabetes Obes Metab. 2023; 10.1111/dom.15011 36775980

[77] •• Manla Y, Almahmeed W. Cardiometabolic clinics: is there a need for a multidisciplinary clinic? Frontiers in Clinical Diabetes and Healthcare. 2022; 10.3389/fcdhc.2022.880468. **In this article, the authors highlighted the benefits of cardiometabolic multidisciplinary clinics in mitigating current practice gaps and improving patient outcomes**. 10.3389/fcdhc.2022.880468 PMC10012126 36992726

[78] •• Okati-Aliabad H, Ansari-Moghaddam A, Kargar S, Jabbari N. Prevalence of obesity and overweight among adults in the Middle East Countries from 2000 to 2020: a systematic review and meta-analysis. J Obes. 2022; 10.1155/2022/8074837. **This systematic review and meta-analysis provides strong evidence that helps better quantify the prevalence of obesity in the Middle East.** 10.1155/2022/8074837 PMC8831052 35154826

[79] Chaabane S, Chaabna K, Abraham A, Mamtani R, Cheema S (2020). Physical activity and sedentary behaviour in the Middle East and North Africa: an overview of systematic reviews and meta-analysis. Sci Rep..

[80] Alsaleh E, Baniyasin F. Prevalence of physical activity levels and perceived benefits of and barriers to physical activity among Jordanian patients with coronary heart disease: a cross-sectional study. Front. Public Health. 2023;10. 10.3389/fpubh.2022.1041428 PMC9846498 36684963

[81] Alsaleh E, Windle R, Blake H (2016). Behavioural intervention to increase physical activity in adults with coronary heart disease in Jordan. BMC Public Health..

[82] Traina MI, Almahmeed W, Edris A, Murat Tuzcu E (2017). Coronary heart disease in the Middle East and North Africa: current status and future goals. Curr Atheroscler Rep..

[83] Bahn R, EL Labban S, Hwalla N (2019). Impacts of shifting to healthier food consumption patterns on environmental sustainability in MENA countries. Sustain Sci..

[84] Dehghan M, Mente A, Zhang X, Swaminathan S, Li W, Mohan V, Iqbal R, Kumar R, Wentzel-Viljoen E, Rosengren A (2017). Associations of fats and carbohydrate intake with cardiovascular disease and mortality in 18 countries from five continents (PURE): a prospective cohort study. The Lancet..

[85] Kazim MN, AbouMoussa TH, Al-Hammadi FA, Al Ali A, Abedini FM, Ahmad FSM, Bazdar MY, Carrick FR, Abdulrahman M (2021). Population awareness of cardiovascular disease risk factors and health care seeking behavior in the UAE. Am J Prev Cardiol..

[86] Aljefree N, Ahmed F (2015). Association between dietary pattern and risk of cardiovascular disease among adults in the Middle East and North Africa region: a systematic review. Food Nutr Res..

[87] Mohammadifard N, Talaei M, Sadeghi M, Oveisegharan S, Golshahi J, Esmaillzadeh A, Sarrafzadegan N (2017). Dietary patterns and mortality from cardiovascular disease: Isfahan cohort study. Eur J Clin Nutr..

[88] Nouri F, Sarrafzadegan N, Mohammadifard N, Sadeghi M, Mansourian M (2016). Intake of legumes and the risk of cardiovascular disease: frailty modeling of a prospective cohort study in the Iranian middle-aged and older population. Eur J Clin Nutr..

[89] Razzak HA, Harbi A, Ahli S (2020). Tobacco smoking prevalence, health risk, and cessation in the UAE. Oman Med J..

[90] Sibai AM, Iskandarani M, Darzi A, Nakkash R, Saleh S, Fares S, Hwalla N (2016). Cigarette smoking in a Middle Eastern country and its association with hospitalisation use: a nationwide cross-sectional study. BMJ Open..

[91] Suwaidi J Al, Zubaid M, El-Menyar AA, Singh R, Asaad N, Sulaiman K, Mahmeed W Al, Al-Shereiqi S, Akbar M, Binali HA Al (2012) Prevalence and outcome of cigarette and waterpipe smoking among patients with acute coronary syndrome in six Middle-Eastern countries. Eur J Prev Cardiol. 19:118–125. 10.1177/1741826710393992 21450616

[92] Amiri P, Mohammadzadeh-Naziri K, Abbasi B, Cheraghi L, Jalali-Farahani S, Momenan AA, Amouzegar A, Hadaegh F, Azizi F. Smoking habits and incidence of cardiovascular diseases in men and women: findings of a 12 year follow up among an urban Eastern-Mediterranean population. BMC Public Health. 2019; 10.1186/S12889-019-7390-0. 10.1186/s12889-019-7390-0 PMC6683328 31382950

[93] Thuresson M, Jarlöv MB, Lindahl B, Svensson L, Zedigh C, Herlitz J (2008). Factors that influence the use of ambulance in acute coronary syndrome. Am Heart J..

[94] Terkelsen CJ, Lassen JF, Nørgaard BL, Gerdes JC, Poulsen SH, Bendix K, Ankersen JP, Gøtzsche LB-H, Rømer FK, Nielsen TT (2005). Reduction of treatment delay in patients with ST-elevation myocardial infarction: impact of pre-hospital diagnosis and direct referral to primary percutanous coronary intervention. Eur Heart J..

[95] Fares S, Zubaid M, Al-Mahmeed W (2011). Utilization of emergency medical services by patients with acute coronary syndromes in the Arab Gulf States. J Emerg Med..

[96] Shehab A, Al-Habib K, Hersi A, Al-Faleh H, Alsheikh-Ali A, Almahmeed W, Suleiman KJ, Al-Motarreb A, Al Suwaidy J, Asaad N (2014). Quality of care in primary percutaneous coronary intervention for acute ST-segment-elevation myocardial infarction: Gulf RACE 2 experience. Ann Saudi Med..

[97] Moafa HN, van Kuijk SMJ, Franssen G, Moukhyer ME, Haak HR (2019). What is known about the quality of out-of-hospital emergency medical services in the Arabian Gulf States? A systematic review. PLoS One..

[98] Collet J-P, Thiele H, Barbato E, Barthélémy O, Bauersachs J, Bhatt DL, Dendale P, Dorobantu M, Edvardsen T, Folliguet T (2021). 2020 ESC Guidelines for the management of acute coronary syndromes in patients presenting without persistent ST-segment elevation: the task force for the management of acute coronary syndromes in patients presenting without persistent ST-segment elevation of the European Society of Cardiology (ESC). Eur Heart J..

[99] AlHabib KF, Sulaiman K, Al Suwaidi J, Almahmeed W, Alsheikh-Ali AA, Amin H, Al Jarallah M, Alfaleh HF, Panduranga P, Hersi A (2016). Patient and system-related delays of emergency medical services use in acute ST-elevation myocardial infarction: results from the Third Gulf Registry of Acute Coronary Events (Gulf RACE-3Ps). PLoS One..

[100] He J, Bellenger NG, Ludman AJ, Shore AC, Strain WD (2022). Treatment of myocardial ischaemia-reperfusion injury in patients with ST-segment elevation myocardial infarction: promise, disappointment, and hope. Rev Cardiovasc Med..

[101] AlHabib KF, Hersi A, AlFaleh H, AlNemer K, AlSaif S, Taraben A, Kashour T, Bakheet A, Al Qarni A, Soomro T (2011). Baseline characteristics, management practices, and in-hospital outcomes of patients with acute coronary syndromes: results of the Saudi project for assessment of coronary events (SPACE) registry. J Saudi Heart Assoc..

[102] Bazzino O, Monaco R, Mario B (2011). Management of acute coronary syndromes in developing countries: acute coronary events—a multinational survey of current management strategies. Am Heart J..

[103] Zubaid M, Rashed WA, Al-Khaja N, Almahmeed W, Al-Lawati J, Sulaiman K, Al-Motarreb A, Amin H, Al-Suwaidi J, Al-Habib K (2008) Clinical presentation and outcomes of acute coronary syndromes in the gulf registry of acute coronary events (Gulf RACE). Saudi Med J. 29:251. 18246236

[104] Alhabib KF, Gamra H, Almahmeed W, Hammoudeh A, Benkheddah S, Al Jarallah M, Al-Motarreb A, Alquraishi M, Sobhy M, Yousif MG (2020). Acute myocardial infarction and acute heart failure in the Middle East and North Africa: study design and pilot phase study results from the PEACE MENA registry. PLoS One..

[105] Alhabib KF, Kinsara AJ, Alghamdi S, Al-Murayeh M, Hussein GA, AlSaif S, Khalaf H, Alfaleh H, Hersi A, Kashour T (2019). The first survey of the Saudi Acute Myocardial Infarction Registry Program: main results and long-term outcomes (STARS-1 Program). PLoS One..

[106] Shaheen S, Wafa A, Mokarab M, Zareef B, Bendary A, Abdelhameed T, Rashwan A, Seleem M, Elmasry M, Abdelhady Y (2020). Presentation, management, and outcomes of STEMI in Egypt: results from the European Society of Cardiology Registry on ST elevation myocardial infarction. Eur Heart J..

[107] Balghith MA (2020). Primary percutaneous coronary intervention facility hospitals and easy access can affect the outcomes of ST-segment elevation myocardial infarction patients. Heart Views..

[108] Shaheen S, Helal A, Anan I (2021). Barriers to the implementation of primary PCI in the management of STEMI in Egypt. Cardiovasc Innov Appl..

[109] Dababo N, Almahmeed N, Edris A, AbdelWareth L, Manla Y, Lee St John T, Al Badarin F (2022). Implementation of high-sensitivity cardiac troponin assay in emergency department increases myocardial infarction diagnosis and utilization of invasive cardiac procedures: an insight from Middle East/Gulf region. Circulation..

[110] Shaheen SM, Saleh AK, Okasha NK, Abdalhamid MA, Fakhry HM, Guindy RR. Implementation of a regional STEMI network in North Cairo (Egypt): impact on the management and outcome of STEMI patients. Glob. Heart. 2023;18. 10.5334/gh.1182 PMC9881442 36760803

[111] Okati-Aliabad H, Ansari-Moghaddam A, Kargar S, Mohammadi M (2022). Prevalence of hypertension and pre-hypertension in the Middle East region: a systematic review & meta-analysis. J Hum Hypertens..

[112] Shara NM (2010). Cardiovascular disease in Middle Eastern women. Nutr Metab Cardiovasc Dis..

